# Bedside-measurement of serum cholinesterase activity predicts patient morbidity and length of the intensive care unit stay following major traumatic injury

**DOI:** 10.1038/s41598-019-46995-y

**Published:** 2019-07-18

**Authors:** Aleksandar R. Zivkovic, Karsten Schmidt, Thomas Stein, Matthias Münzberg, Thorsten Brenner, Markus A. Weigand, Stefan Kleinschmidt, Stefan Hofer

**Affiliations:** 1 Department of Anesthesiology, Heidelberg University Hospital, Heidelberg, Germany; 2 Department of Anaesthesia, Intensive Care Medicine and Pain Therapy, BG Trauma Center Ludwigshafen/Rhine, Ludwigshafen, Germany; 3 Department of Trauma and Orthopaedic Surgery, BG Trauma Center Ludwigshafen/Rhine, Ludwigshafen, Germany; 4 Clinic for Anesthesiology, Intensive Care, Emergency Medicine I and Pain Therapy, Westpfalz Hospital, Kaiserslautern, Germany

**Keywords:** Predictive markers, Trauma, Predictive markers, Acute inflammation, Acute inflammation

## Abstract

Major traumatic injury (MTI), a life-threatening condition requiring prompt medical intervention, is associated with an extensive inflammatory response often resulting in multiple organ dysfunction. Early stratification of trauma severity and the corresponding inflammation may help optimize resources at the intensive care unit (ICU). The cholinergic system counters inflammation by quickly modulating the immune response. Serum cholinesterase (butyrylcholinesterase, BChE) is an enzyme that hydrolyses acetylcholine. We tested whether a change in the BChE activity correlates with the morbidity and the length of ICU stay. Blood samples from 10 healthy volunteers and 44 patients with MTI were gathered at hospital admission, followed by measurements 12, 24 and 48 hours later. Point-of-care approach was used to determine the BChE activity. Disease severity was assessed by clinical scoring performed within 24 hours following hospital admission. BChE activity, measured at hospital admission, showed a significant and sustained reduction and correlated with disease severity scores obtained 24 hours following admission. BChE activity, obtained at hospital admission, correlated with the length of ICU stay. Bedside measurement of BChE activity, as a complementary addition to established procedures, might prove useful in the primary assessment of the disease severity and might therefore optimize therapy in the ICU.

## Introduction

Major traumatic injury (MTI) is an acute and potentially life-threatening condition which requires immediate and intensive medical intervention^1^. MTI has been associated with prolonged stay in the emergency department^2^ and increased mortality^3,4^. Literature suggests that patients with severe injury are associated with a mortality risk of 10–26%^5^. MTI patients require intensive medical resources, expensive medical interventions and show prolonged stay in the intensive care unit (ICU)^6^. Early assessment of MTI severity and prevention of complications associated with this acute medical condition play a significant role in optimizing treatment and resources required for these patients during their stay at the ICU.

MTI triggers a systemic inflammatory response^7,8^ which shows both pro- and anti-inflammatory actions^9^. The two modes of the immune response often interchange quickly in the initial and critical phase following traumatic injury. Extreme forms of the pro-inflammatory response in immunocompetent patients might trigger a state of shock, eventually resulting in multiple organ dysfunction/failure syndrome^10^. Therefore, patients with MTI require meticulous monitoring of their inflammatory status in order to prevent progression to an irreversible state of shock. However, early assessment of the magnitude and the extent of the inflammatory response following traumatic injury remains a challenge for modern emergency and intensive care medicine.

The cholinergic system has been shown to act anti-inflammatory by modulating the immune system via non-neuronal acetylcholine^11–14^. Serum cholinesterase (butyrylcholinesterase, BChE) is an enzyme that hydrolyses acetylcholine. BChE is synthesized in the liver and is abundant in blood^15^. Altered BChE enzyme activity has been suggested to correlate with the emerging inflammation^16^. A change in the BChE activity, observed as early as 1 hour following a modest traumatic injury, could indicate an emerging inflammatory response^17^, providing a marker for early detection. Bedside measurement of the BChE activity provides a quick assessment of the cholinergic activity upon an inflammatory response.

Various factors (i.e. age, renal failure, sepsis, respiratory failure), clinical scores (ISS, GCS) and laboratory measurements (i.e. haemoglobin, lactate, base excess, platelet count) have been used to estimate the course and the magnitude of the inflammatory reaction and the resulting disease severity of the patients with traumatic injury^18^. Predicting the length of the ICU stay might play an important role in staff and resources management for these patients^19–21^. The conventional clinical scores (e.g. Acute Physiology And Chronic Health Evaluation II - APACHE II; Simplified Acute Physiology Score II - SAPS II; Sequential Organ Failure Assessment – SOFA; Injury Severity Score - ISS) are easy to use, well established and efficient in assessing the disease severity and the length of the ICU stay. However, these tests often cannot be assessed immediately following hospital admission. Moreover, in the case of SOFA, even when used in its abbreviated from, adapted for a quick disease severity assessment (quick SOFA)^22^ an occurrence or progression of an organ dysfunction/failure within 24 hours is required for the clinical scores to be calculated and interpreted, further postponing clinical feedback^23^. The initial 24 h after hospital admission is the time window where the primary resuscitation, as well as resource management take place, however indicators of the potential emergence of an inflammatory response are often lacking.

Here we tested whether a change in the BChE activity, measured upon their hospital admission in patients with MTI, could be associated with the duration and the complexity of the intensive care treatment and therefore be used for early risk stratification.

## Results

Fifty patients with major traumatic injury were included in this study. Out of 50 recruited patients, 3 patients received a massive blood transfusion within the first 48 hours of the observation period. In addition, two of these three patients also had a documented extensive liver function disruption. Further 3 patients were presented with a manifest inflammation. These 6 patients were therefore excluded from the study, resulting in a cohort of 44 patients. Basic demographic data, disease severity and the length of the intensive care unit stay are shown in the Table 1.

**Table 1:** Characteristics of the patients with major traumatic injury.

Patient characteristics		
number of patients		44
age		55 (32–72)*
gender (male/female)		33/11
**Injury**		
ISS		27 (20–34)*
**Disease severity**		
28-day survivors		43
Length of ICU stay		9 (5–16)*
scores obtained 24 h following hospital admission	APACHE II	17 (12–23)*
	SAPS II	41 (28–47)*
	SOFA	7 (5–10)*

*Median (interquartile range).

ISS – Injury Severity Score; ICU – Intensive Care Unit; APACHE II - Acute Physiology And Chronic Health Evaluation II; SAPS II - Simplified Acute Physiology Score; SOFA - Sequential Organ Failure Assessment.

### An immediate and sustained reduction in BChE activity is observed in patients following MTI

**Figure 1:**
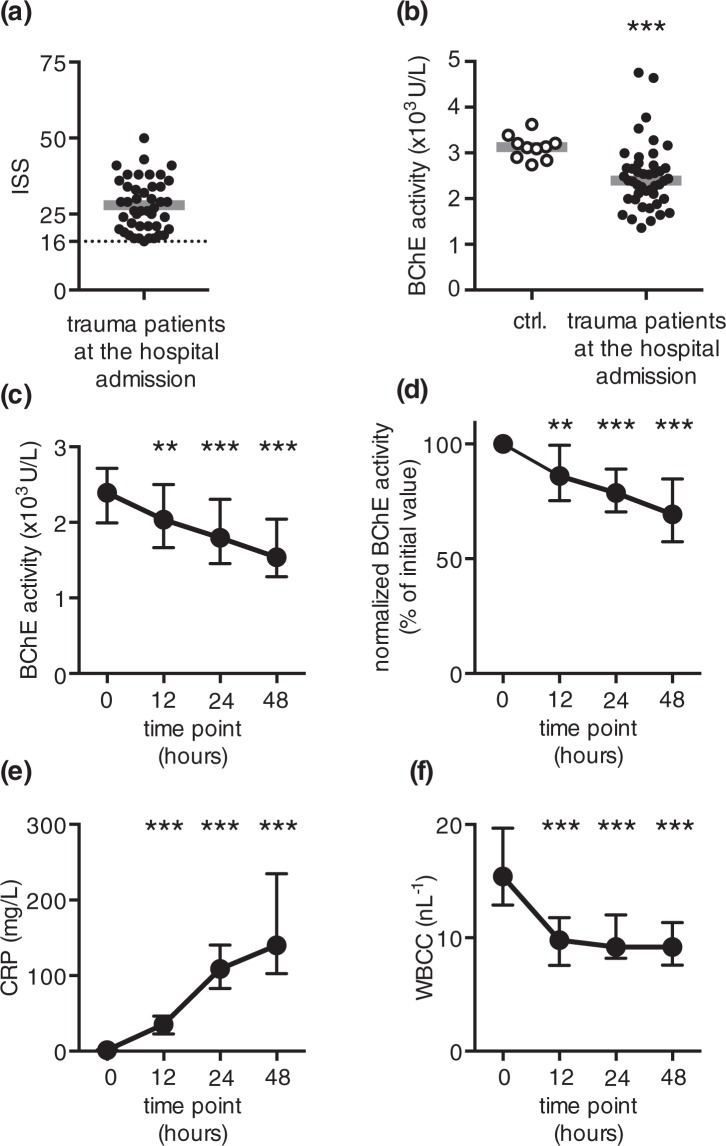
An immediate and sustained reduction in BChE activity occurs following major traumatic injury. **(a)** Scatter plot represents ISS scores obtained from trauma patients upon arrival to the emergency department **(b)** Data points represent BChE activity measured from 10 healthy volunteers (ctrl., open circles) and 44 major trauma patients at the hospital admission (closed circles). (**c**) The activity of BChE measured in injured patients 12, 24 and 48 hours following hospital admission significantly decreased, when compared to the initial measurement at hospital admission. (**d**) BChE activity data shown in (**c**), normalized to the initial value obtained at hospital admission. (**e**) Concurrent measurement of CRP activity revealed a continuous increase starting 12 h after hospital admission. (**f**) The initial elevation in WBCC decreased 12 hours later and remained constant throughout the observation period. Grey lines in (**a,b**) are medians. Error bars are interquartile range. **p < 0.01; ***p < 0.001 (Friedman test followed by Dunn’s multiple comparisons test); ISS – Injury Severity Score; ctrl. – control (healthy volunteers); CRP – C-reactive protein; WBCC – white blood cell count.

Injury severity score (ISS) was used to assess the severity of the trauma in MTI patients **(**Fig. 1a, Table 1). The enzymatic activity of the BChE, measured from MTI patients at the hospital admission showed a marked reduction as compared to that of healthy volunteers **(**Fig. 1b, Supplementary File 1**)**. The measured BChE activity of trauma patients remained reduced during the 48-hour observation period (Fig. 1c, Supplementary File 1). To quantify the amount of the enzyme reduction and to compensate for the inter-individual variability of the initially measured BChE activity, we normalized the obtained measurements to the initial value. The analysis of the normalized BChE activity revealed a comparable result: a significant and sustainable reduction of the normalized BChE activity, starting 12 hours following hospital admission (Fig. 1d, Supplementary File 1). The observed reduction of the normalized enzyme activity suggests a prompt and sustained BChE reduction upon traumatic injury, regardless of the initial enzyme activity. Concurrently measured C-reactive protein (CRP) activity revealed a comparable and sustainable increase in these patients, starting 12 hours following hospital admission (Fig. 1e, Supplementary File 1). Severely injured patients showed elevated white blood cell count (WBCC) at the hospital admission. WBCC decreased 12 h later and remained stable above the normal laboratory range during the 48-hour observation period (Fig. 1f, Supplementary File 1).

### The initially measured BChE activity negatively correlates with disease severity scores obtained within 24 hours following ICU admission

**Figure 2:**
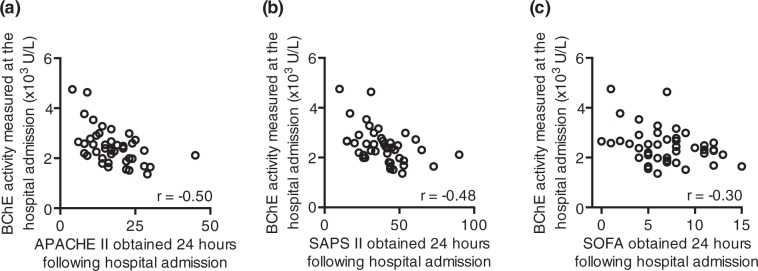
The BChE activity measured upon arrival to the hospital correlates with the disease severity scores obtained 24 hours later. Scatter diagrams represent a correlation of the BChE activity, measured from major trauma patients at the hospital admission and the APACHE II (**a**), SAPS II (**b**) and SOFA (**c**) scores obtained 24 hours later. r – Spearman correlation coefficient; APACHE II - Acute Physiology And Chronic Health Evaluation II; SAPS II - Simplified Acute Physiology Score; SOFA - Sequential Organ Failure Assessment.

We used clinical disease severity scores to assess the disease severity of the trauma patients. Disease severity of these patients was assessed by using APACHE II, SAPS II and SOFA scoring within 24 hours following hospital admission (Table 1). Next, we tested whether the initially measured BChE activity correlated with the disease severity, documented up to 24 hours later in MTI patients. Indeed, the BChE activity, measured at the hospital admission, negatively correlated with APACHE II (r = −0.50; moderately strong negative correlation; r - Spearman correlation coefficient; Fig. 2a), SAPS II (r = −0.48; moderately strong negative correlation; r - Spearman correlation coefficient; Fig. 2b), as well as SOFA score (r = −0.30; weak negative correlation; r - Spearman correlation coefficient; Fig. 2c), obtained within 24 hours following ICU admission. In contrast, CRP and WBCC, measured at the hospital admission, showed no correlation with the disease severity scores (data no shown).

### The BChE activity obtained upon hospital arrival negatively correlates with the length of the ICU stay

**Figure 3:**
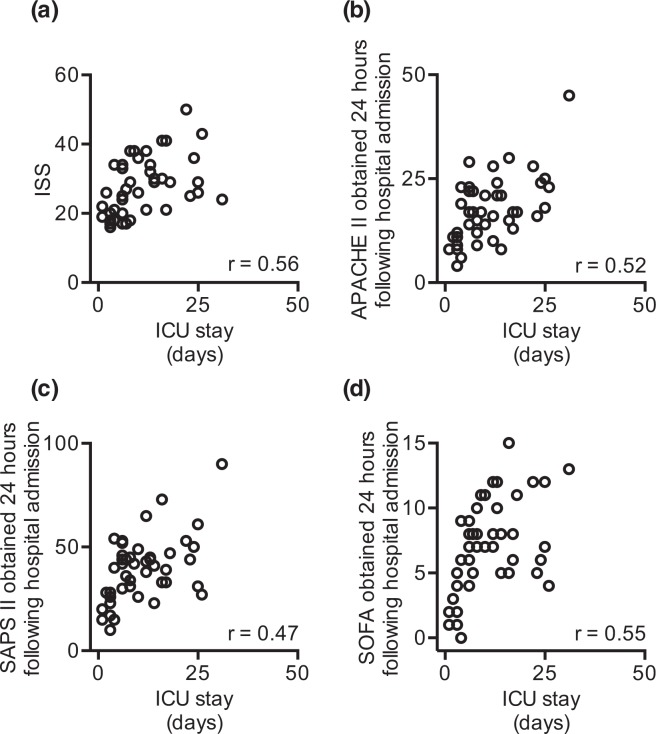
Disease severity scores correlate with the length of ICU stay following major traumatic injury (MTI). (**a**) The obtained ISS score correlates with the length of the ICU stay of severely injured patients. **(b)** APACHE II, **(c)** SAPS II and **(d)** SOFA scores obtained 24 hours after the hospital admission following MTI correlate with the length of ICU stay. r – Spearman correlation coefficient; ICU – intensive care unit; ISS – injury severity score; APACHE II - Acute Physiology And Chronic Health Evaluation II; SAPS II - Simplified Acute Physiology Score; SOFA - Sequential Organ Failure Assessment.

**Figure 4:**
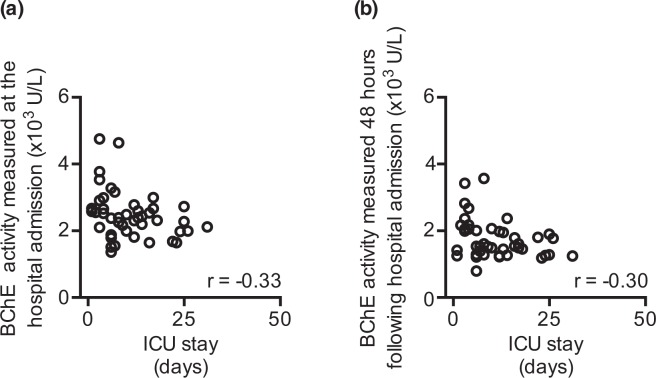
The activity of BChE measured at hospital admission correlates with the length of ICU stay in patients with major traumatic injury. The activity of BChE measured from trauma patients at hospital admission (**a**) and 48 hours later (**b**) correlates with their length of the ICU stay. r – Spearman correlation coefficient; ICU – intensive care unit.

We asked whether the obtained ISS score correlated with the length of the ICU stay. The analysis revealed that the ISS score directly correlated with the length of the ICU stay (r = 0.56; moderately strong positive correlation; r - Spearman correlation coefficient; Fig. 3a). We next evaluated whether clinical disease severity scores correlate with the length of the ICU stay, when obtained within 24 hours following hospital admission. As expected, we observed a correlation between the APACHE II (r = 0.52; moderately strong positive correlation; r - Spearman correlation coefficient; Fig. 3b), SAPS II **(**r = 0.47; moderately strong positive correlation; r - Spearman correlation coefficient; Fig. 3c) as well as the SOFA score (r = 0.55; moderately strong positive correlation; r - Spearman correlation coefficient; Fig. 3d) and the length of the ICU stay. In contrast, CRP did not correlate with the length of the ICU stay, regardless of the measurement sample timepoint within the 24 hours following hospital admission (data not shown). Finally, the activity of the BChE, when measured at the hospital admission (r = −0.33; weak negative correlation; r – Spearman correlation coefficient; Fig. 4a) and 48 hours later (r = −0.30; weak negative correlation; r – Spearman correlation coefficient; Fig. 4b) negatively correlated with the length of the ICU stay.

## Discussion

In this study we report a marked reduction in the BChE activity, observed at hospital admission in patients with major traumatic injury. Furthermore, we report that a change in the enzymatic activity of the BChE following major traumatic injury correlates with the increased conventional inflammatory biomarkers. This finding is in line with our previous reports, suggesting that the BChE might be a useful marker for early detection of systemic inflammation^17^. Finally, the BChE activity, measured at the hospital admission correlates with the length of the ICU stay.

An increase in the CRP activity reliably identifies an ongoing inflammation rendering this biomarker a gold-standard in modern medicine^24–27^. However, a significant increase in CRP activity, associated with the ongoing inflammatory process occurs with a time delay of 12 hours, reaching peak activity 2–4 days following an inflammatory challenge^25,28–32^. WBCC has been shown to be a rather unspecific parameter for identifying an ongoing inflammation with variable onset and peak activity times^33^. Our data supports the observation that the leukocyte count should not be used as a single parameter for the diagnosis of systemic inflammation^28^.

Our previous study reported a significant reduction in the BChE activity detectable as early as one hour following mild traumatic injury, rendering this assay quick and sensitive tool for detecting emerging systemic inflammation^17^. Moreover, our present data suggests that by using a point-of-care approach, the time from the initial patient contact until the first laboratory result could be further shortened.

The pathophysiologic mechanism behind the BChE activity change and the systemic inflammation presumably involves the non-neuronal anti-inflammatory activity of the cholinergic system^12,14,34,35^. Pro-inflammatory mediators such as interleukin (IL)-1β, IL-6, IL-18 and tumor necrosis factor (TNF), activate the cholinergic system via vagal nerve stimulation, to inhibit their pro-inflammatory activity in an anti-inflammatory feedback manner^11^. The activity of the serum cholinesterase could serve as a surrogate readout of the non-neuronal cholinergic activity upon the inflammatory response. However, the question of how the change in the activity of the serum cholinesterase relates to the level of the non-neuronal acetylcholine in serum and the systemic immune response remains unknown.

Medical treatment of patients with MTI is immediate, complex and challenging, often requiring the continuous presence of skilled staff and the utilization of specialized medical equipment, placing an often excessive burden on hospital resources^20,36^. Primary assessment of the disease severity plays a crucial role in the initial decision-making process for the treatment and management of these patients in the ICU^20^. ICU staff need initial medical diagnostics results (e.g. radiography, computer tomography, sonography), laboratory tests and clinical disease severity scores to arrange and optimize essential and often complex procedures for patients with MTI. A large number of these primary procedures are readily accessible in specialized trauma centres. However, these procedures often remain a challenge in the resource-limited settings. Certain test results and procedures are only available 24 hours or more following hospital admission (in hospitals located in rural or remote areas), or unavailable in some clinics (e.g. computer tomography). Missing or delayed initial test results (e.g. delayed diagnostics results, missing disease severity scores) from patients with MTI is disadvantageous, particularly during the primary resuscitation phase when critical decisions, including any early decision to transfer patients to the next suitable clinic, have to be made immediately^37^. Here, we observed that the BChE activity, when measured at the time point of patient admission to the hospital, correlates with the disease severity scores obtained within 24 hours. Disease severity scores, which are broadly used for the standardized clinical categorization of the ICU patients, have been shown to correlate well with patient outcome and the length of hospital stay^19,38–40^. Length of the ICU stay is an important parameter in the assessment of the quality and efficacy of the medical care^38^ as well as a benchmark for process optimization in the ICU^21^. Therefore, bedside measurement of the BChE activity could offer an initial assessment of the patient status and outcome and, if combined with conventional procedures, might help to optimize primary assessment and therapy of severely injured patients.

Only one patient in this study cohort did not survive the observation period of 28 days following MTI. Therefore, it was not feasible to use mortality rate as a primary outcome for this study, nor to draw any conclusions regarding the ability of the bedside BChE activity measurement in predicting patient survival. A large multicentre study would be required to assess whether BChE activity measurement can predict patient survival following MTI.

We must point out several limitations of the BChE activity assay in the ICU. Firstly, since BChE is synthesized in liver, this assay is unsuitable for patients with major liver dysfunction and the authors currently possess no data for a reliable interpretation of the BChE activity in these patients. Secondly, enzyme BChE is present in readily available blood products and caution should be used when interpreting BChE activity results from patients who have received blood transfusions. Patients subjected to a massive blood transfusion were excluded from this study (as described in the exclusion criteria of the Methods section). Thirdly, the BChE activity assay cannot identify the cause of the systemic inflammation (sterile inflammation vs. pathogen-induced infection). Therefore, BChE assay should be used in a combination with other available methods to identify inflammation.

In conclusion, decreased BChE enzymatic activity, measured at the hospital admission, correlates with elevated inflammatory biomarkers and with clinical disease severity scores obtained from patients with major traumatic injury. Moreover, the depression of BChE activity correlates with the length of the ICU stay, identifying this assay as a useful biomarker in the risk stratification and initial evaluation of disease severity in these patients. Therefore, prompt assessment of the systemic immune response with an immediate, rapid and affordable bedside measurement of the BChE activity might improve risk evaluation, primary patient assessment and help optimize ER and ICU therapy in patients with MTI.

## Methods

### Study design

This observational cohort study (ClinicalTrials.gov Identifier NCT02691650) was performed at the emergency room (ER) and the intensive care unit of the BG Trauma Center Ludwigshafen/Rhine over nine months starting in April 2016. The Ethics Committees of the Medical Faculty at the Heidelberg University (file numbers: S-196/2014 and S-391/2015) and the Rheinland-Pfalz Medical Board (file number: 837.539.15/10307) approved this study. All methods were performed in accordance with the relevant guidelines and regulations. All patients and/or their legal guardian/s, as well as all healthy volunteers gave informed consent. Patients with an MTI were admitted to the emergency room^41^. The trauma severity was assessed by using the Injury Severity Score (ISS)^4^. Patients with an ISS ≥ 16 were included in the study. The following exclusion criteria were defined for this study: a manifested pre-existing inflammation, documented with C-reactive protein (CRP) > 10 mg/L^25^ or white blood cell count (WBCC) > 10 nL^−1^ at hospital admission; a disrupted liver function with positive results in three or more of the following tests: aspartate aminotransferase (ASAT) > 100 U/L, alanine aminotransferase (ALAT) > 100 U/L, gamma glutamyl transferase (GGT) > 100 U/L, total bilirubin >2 mg/dL, and international normalized ratio (INR) > 1.3; a massive blood transfusion with more than 5000 ml of packed red blood cells (PRBC) or fresh frozen plasma (FFP) within the 48 hours of the observation period.

### Measurements

Blood samples used in this study were gathered at the time point of the patient admission to the emergency room of our hospital, and 12, 24 and 48 hours later. Serum cholinesterase enzyme activity was measured by using the point-of-care testing (POCT) device ChE Check (Securetec Detektions-Systeme AG, Neubiberg, Germany; *In-Vitro*-Diagnostics Guideline 98/79/EG; DIN EN ISO 18113-2 and -3) according to the manufacturer’s instructions, as previously described^16^. In brief, a minimal amount of blood (10 µl) was required for the POCT analysis of BChE activity, providing rapid quantification of BChE activity in human blood without any pretreatment of the samples. Enzyme activity was determined by measuring the production of thiocholine indirectly from the hydrolysis of the specific substrate s-butyrylthiocholine iodide. Thiocholine reacts with 5,5′-dithio-bis-2-nitrobenzoic acid (DTNB, Ellman’s reagent) as a chromogenic reagent, producing the yellow 5-thio-2-nitrobenzoate anion (TNB, Ellman’s anion). The TNB production was monitored at 470 nm. Enzyme activity is expressed as kU/L. Conventional inflammation biomarkers (CRP and WBCC) were analysed according to the standardized protocols of the clinic laboratory. Disease severity of injured patients was assessed at the ICU with APACHE II (Acute Physiology And Chronic Health Evaluation II), SAPS II (Simplified Acute Physiology Score) SOFA (Sequential Organ Failure Assessment) and ISS (Injury Severity Score) scores measured within the first 24 hours following hospital admission. APACHE II is a disease severity classification scoring system applied within 24 hours following patient admission to the ICU. APACHE II is a nominal score ranging from 0 to 71. The APACHE II score represents a sum of the following measurements: body temperature (0–4 points), mean arterial pressure (0–4 points), heart rate (0–4 points), respiratory rate (0–4 points), A-aPO2 (FiO2 > 50%) or PaO2 (FiO2 < 50%) (0–4 points), arterial pH or HCO3 (0–4 points), serum sodium (0–4 points), serum potassium (0–4 points), serum creatinine (0–8 points), hematocrit (0–4 points), WBCC (0–4 points), Glasgow Coma Scale (15-GCS), age (0–6 points), chronic health problems (0–5 points). SAPS II, ranging from 0 to 163, is a clinical score based on the sum of points obtained from the following measurements: age (0–18 points), heart rate (0–11 points), systolic blood pressure (0–13 points), body temperature (0–3 points), Glasgow Coma Scale (0–26 points), PaO2/FiO2 (0–11 points), blood urea nitrogen (0–10 points), urine output (0–11 points), serum sodium (0–5 points), serum potassium (0–3 points), bicarbonate (0–6 points), bilirubin (0–9 points), WBCC (0–12 points), chronic disease (0–17 points), type of admission (0–8 points). SOFA is a disease severity score consisting of 6 variables, representing organ systems (respiration, coagulation, liver, neurological, cardiovascular and renal). Each organ system is assigned a point value (0–4), comprising a SOFA score range from 0 to 24. ISS is body region based nominal scale ranging from 1 to 75. Body regions are grouped into six: head or neck, face, chest, abdominal or pelvic contents, extremities or pelvic girdle, and external. The severity of the injuries is scored by using Abbreviated Injury Score (AIS) ranging from 0 to 6. The ISS score is calculated as the sum of the squares of the highest AIS scores for the three most severely injured body regions. All patients completed the 28-day-survival observation period.

### Statistical analysis

The patient data and the measurements were electronically gathered and stored using Excel (Microsoft Corp., Redmond, WA). Data analysis was performed by using GraphPad Prism 6 for Mac (GraphPad Software, La Jolla California, USA, http://www.graphpad.com). The study size was calculated by using Cohen’s *d*-test (*d* = 0.57; power 80%, significance level alpha = 5%). D’Agostino and Pearson omnibus normality test was used to verify the Gaussian distribution of the study groups. Data are presented as median with interquartile range (IQR). Statistical significance between the patient groups was tested using either the Mann-Whitney or the Friedman test followed by Dunn’s multiple comparisons test. Correlation was tested using Spearman correlation test. A p value < 0.05 indicated statistical significance.

### Ethics approval and consent to participate

The Ethics Committees of the Medical Faculty at the Heidelberg University (file numbers: S-196-2014 and S-391/2015) and the Rheinland-Pfalz Medical Board (file number: 837.539.15/10307) approved this study.

## Supplementary information

Supplementary file 1

## Data Availability

The datasets generated during and/or analysed during the current study are available from the corresponding author on reasonable request.
