# Supplementary material for: Bedside-measurement of serum cholinesterase activity predicts patient morbidity and length of the intensive care unit stay following major traumatic injury
Source: Sci Rep. 2019 Jul 18;9:10437. doi: 10.1038/s41598-019-46995-y (PMC6639389; doi:10.1038/s41598-019-46995-y)
Supplement: Supplementary file 1 [file 41598_2019_46995_MOESM1_ESM.docx]

**Title:**

Bedside-measurement of serum cholinesterase activity predicts patient morbidity and length of the intensive care unit stay following major traumatic injury

**Authors:**

Aleksandar R. Zivkovic, MD, Karsten Schmidt, MD, Thomas Stein, MD, Matthias Münzberg, MD, Thorsten Brenner, MD, Markus A. Weigand, MD, Stefan Kleinschmidt, MD, Stefan Hofer, MD

| **Supplementary file 1** | | | | | | |
| --- | --- | --- | --- | --- | --- | --- |
| **Results summary of the Figure 1** | | | | | | |
|  | | | | | | |
|  |  |  | hospital admission | 12 hours | 24 hours | 48 hours |
| Healthy volunteers | BChE activity  (x10^3^ U/L) | 3.1 (2.9-3.3) | n.a. | n.a. | n.a. | n.a. |
| Patients | BChE activity  (x10^3^ U/L) | n.a. | 2.4 (2.0-2.7) | 2.0 (1.7-2.5) | 1.8 (1.5-2.3) | 1.5 (1.3-2.0) |
|  | normalized BChE activity  (% of initial value) | n.a. | n.a. | 86 (75-99) | 79 (70-89) | 69 (57-85) |
|  | CRP (mg/L) | n.a. | 1 (0-2) | 35 (23-46) | 109 (83-140) | 140 (103-235) |
|  | WBCC (nL^-1^) | n.a. | 15 (13-20) | 9.8 (7.6-12.0) | 9.2 (8.2-12.0) | 9.2 (7.6-11.0) |
| BChE – butyrylcholinesterase; CRP – C-reactive protein; WBCC – white blood cell count; n.a. – not applicable; data are presented as median with interquartile ratio. | | | | | | |
